# A Panel Analysis of the Strategic Association Between Information and Communication Technology and Public Health Delivery

**DOI:** 10.2196/jmir.2242

**Published:** 2012-10-22

**Authors:** Sarah Jinhui Wu, Wullianallur Raghupathi

**Affiliations:** ^1^Management SystemsThe Gabelli School of BusinessFordham UniversityBronx, NYUnited States; ^2^Graduate School of BusinessInformation and Communication SystemsFordham UniversityNew York, NYUnited States

**Keywords:** Association, Correlation, Information and communication technologies (ICTs), Panel data, Public health delivery

## Abstract

**Background:**

In this exploratory research, we use panel data analysis to examine the correlation between Information and Communication Technology (ICTs) and public health delivery at the country level.

**Objective:**

The goal of this exploratory research is to examine the strategic association over time between ICTs and country-level public health.

**Methods:**

Using data from the World Development Indicators, we construct a panel data set of countries of five different income levels and look closely at the period from 2000 to 2008. The panel data analysis allows us to explore this dynamic relationship under the control for unobserved country-specific effects by using a fixed-effects estimation method. In particular,, we examine the association of five ICT factors with five public health indicators: adolescent fertility rate, child immunization coverage, tuberculosis case detected, life expectancy, and adult mortality rate.

**Results:**

First, overall ICTs’ factors substantially improve a country’s public health delivery on the top of wealth effect. Second, among all the ICTs’ factors, accessibility is the only one that is associated with improvements in all aspects of public health delivery, while the contributions from the usage, quality, and applications are negligible. ICTs’ accessibility factor is associated with a considerable extension to life expectancy and reduced adult mortality rate. Third, all entity-specific factors are significant in each model, indicating that countries’ economic development level does influence their public health delivery.

**Conclusions:**

Our results indicate that ICT accessibility has a strong association with effective delivery of public health. There are others, but the key strategic applications are eHealth and mHealth. The findings of this study will help government officials and public health policy makers to formulate strategic decisions regarding the best ICT investments and deployment. For example, the study shows that providing accessibility should be a critical focus.

## Introduction

Despite the slow diffusion of information and communication technology (ICT) to support public health delivery worldwide as well as the high cost of implementation, global health policy makers and public health officials must focus on key strategic applications of ICTs that deliver high-quality public health care at lower costs. ICTs can be characterized by such factors as access, quality, and applications among others. Specifically, the applications may include such categories as eHealth, mHealth, situation awareness systems, and “smart” health systems [1-13] among others. eHealth refers to the use of ICTs such as computers, mobile phones, and satellite and wireless communication systems for health information and services [1,6,12,14]. mHealth indicates the use of mobile communications such as personal digital assistants (PDAs) and mobile phones to provide remote health information and services [15]. Situation awareness systems enable us to prepare for and respond to disease outbreaks and other public health emergencies by automating the surveillance processes and the collection, analysis, and reporting of the health data [5]. Smart health systems refer to the use of ICTs for better connection, higher-quality data, and real-time analysis of the data. Doctors, patients, insurers, and government officials can share health information seamlessly and efficiently. 

Understanding the association between ICTs and the nature of public health delivery is important [16,17] since ICTs have the potential to accelerate the improvement in public health. ICTs provide the infrastructure and resources for the development of large-scale population-level applications such as health information networks, surveillance systems, and telemedicine. ICTs can improve health outcomes and combat diseases. For example, in rural Niger, following the introduction of a radio ambulance system, the number of emergency evacuations from outlying health centers to the district hospital rose from 10 to 197. Good communications and information sharing help to deliver diagnostic information and drugs and to spread information on reproductive health and communicable diseases. Through the Global Media acquired immunodeficiency syndrome (AIDS) initiative, more than 50 broadcast networks are promoting AIDS prevention messages. In the fight against malaria, satellite monitoring identifies and targets mosquito breeding areas for control [18]. Other examples of ICT-based systems at work (reported in [6]) include telemedicine networks in Bangladesh; e-pharmacy projects in Malaysia; low-cost sustainable electronic medical records for human immunodeficiency virus infection (HIV)/AIDS patients in Kenya; and web-based communication tools to address material and child deaths in Peru [6].

In addition, public health informatics (PHI) was defined as the “systematic application of information and computer science and technology to public health practice, research and learning that integrates public health and information technology” [19]. PHI needs to use a “systematic and informed approach to the application of information science and technology in order to take full advantage of its potential to enhance and facilitate public health activities” [20].

Given the potential linkage between ICTs and public health, the purpose of this paper is to empirically evaluate the two related research questions: (1) Do ICTs have a strong association with a country’s public health delivery indicators over time?; (2) Which of the key ICT factors are associated with improvement in public health delivery?

**Figure 1:**
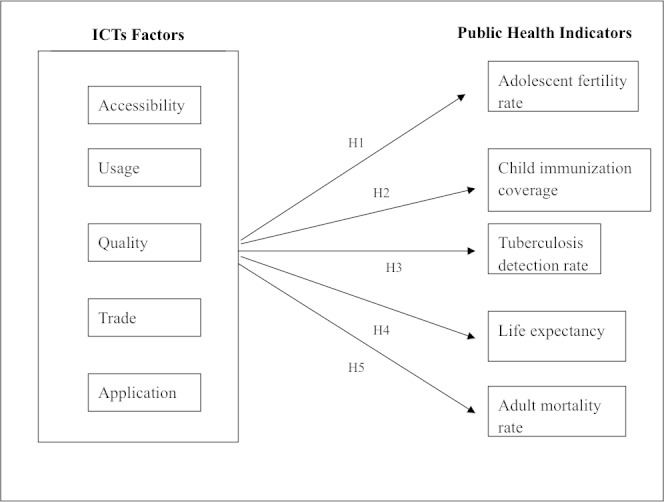
Conceptual framework.

The framework in this paper is an adaptation of the conceptual framework developed in an earlier paper (see [17] for comprehensive literature review) as well as the publications at the International Telecommunication Union (ITU) [8-10], World Health Organization (WHO) [13,18], and World Bank [21]. We also draw on the substantive health information technology literature [17] and the recent special issues of Journal of the Association for Information Systems (JAIS) [22] and Journal of Strategic Information Systems (JSIS) [23]. Both anecdotal evidence and various surveys indicate a potential association between ICTs and country-level public health delivery as previously discussed. As such, we propose a positive association between ICTs (independent variables) and public health variables (dependent variables) (Figure 1). The ICT factors are derived from the World Bank ICT at-a-Glance Tables [24] that include accessibility, quality, usage, affordability, trade, and application. We chose for our macro-level study the significant aggregate indicators related to public health [25] that contain adolescent fertility rates, child immunization coverage, tuberculosis detection rates, life expectancy, and adult mortality rates.

While proposing these associations we recognize the reality that high-income countries would have better ICT infrastructure and better public health. That is, ICTs may not have an association with global public health delivery; wealth may provide the association. To obviate this possibility, the wealth effect is controlled so as to obtain accurate and reliable estimates. We discuss next the public health indicators in context of the hypotheses.

### Public Health Indicators

#### Adolescent Fertility Rate

A high number for this indicates that fewer children are going to school; additionally, early childbirth has its own complications with less access to post-childbirth health care. ICTs, in aggregate can help in educating this population to take preventive steps, assist in delivering health information via eHealth and mHealth, and generally assist in reducing the rate. Therefore, we formulate hypothesis 1 as: H1. Information and communication technology (ICT) is associated with a reduction in the adolescent fertility rate over time.

#### Child Immunization Rate

A high number for this variable would indicate better quality preventive health care and thereby improved public health. The different ICT factors can influence the child immunization rate in positive ways such as enabling the communication and dissemination of immunization information, educating the public about the benefits of immunization in reducing instances of preventable diseases, and facilitating the delivery of immunization by health workers via setting up a database of records, tracking, and generating reminders for follow-up. So we formulate hypothesis 2 as: H2. Information and communication technology (ICT) is associated with an increase in child immunization coverage over time.

#### Tuberculosis Detection Rate

This represents other diseases such as malaria, HIV/AIDs, and other in terms of detection, monitoring, and prevention. ICTs can help in several ways to increase the detection rate. For one, advanced ICTs can help in the analysis of the blood work and the intakes from the tests and procedures. The collected information can be organized and analyzed to gain insight to make informed decisions regarding the disease status. Additionally, ICTs can help communicate the results and follow-up guidelines to the patients (eg, via texting or mobile phone calls) for prompt action. Therefore, hypothesis 3 is formulated as: H3. Information and communication technology (ICT) is associated with an increase in the tuberculosis case detection rate over time.

#### Life Expectancy

A key goal of development as well as health is to increase the life expectancy of a country’s citizens so human beings can achieve their full potential. The different ICT factors can contribute overall to the achievement of this goal via providing health-related information to the public, for example on hygiene, sanitation, and other good practices on living. Additionally, health workers can be provided with ICT-enabled accessories to educate the public; identify, gather, and document information about health status; and to perform follow-up activities. Educating the public is necessary to improved public health, and ICTs can play a critical role in this regard. We formulate hypothesis 4 as follows: H4. Information and communication technology (ICT) is associated with an increase in the life expectancy over time.

#### Adult Mortality Rate

This again represents the overall human condition status of a country. The goal is to reduce the adult mortality rate. This is possible by focusing on good public health and preventive measures. ICTs can play a significant role in many ways including education for preventive care, delivery of health care via telemedicine, etc, and the monitoring of health conditions such as spread of diseases, pandemics, etc, via the use of mHealth, Internet, and radio and television. By reducing the adult mortality rate, a country can take advantage of a healthy adult population to engage in productive work. Therefore, hypothesis 5 is formulated as such: H5. Information and communication technology (ICT) is associated with a reduction in the adult mortality rate over time.

## Method

In a prior cross-sectional study of 200 countries using secondary data from World Bank, it was found that in most cases cumulated investment in ICTs is strongly associated with a country’s public health delivery [17]. Yet, the nature of the dataset limited the ability to make strong inferences between the two even though the wealth effect was controlled in the analysis. To estimate the extent of the association between ICTs and improved country public health delivery, this study looks at a panel data set of countries representing five different income levels from 2000 to 2008. It has been argued that this association also leads to economic growth and poverty reduction. The panel data set allows us to explore these dynamic relationships while controlling for unobserved country-specific effects. We use a fixed-effects estimation method to control for unobserved country factors within an income level that may be correlated with countries’ public health delivery. We used this method because any unobserved country factor is likely to bias estimated coefficients in a traditional regression model. This method also allows us to examine the wealth effect of different income levels on countries’ public health delivery. Overall, this panel analysis delineates the impacts of income level and ICT factors on countries’ public health delivery, thus providing more accurate evaluation of the contribution of ICTs to public health delivery.

### Data Collection and Measurements

The data used to test our hypotheses came from two World Bank databases for more than 200 countries during 2000 to 2008. The World Bank ICT at-a-Glance database [24] classifies the major ICT sector performance variables into six factors, namely accessibility, usage, quality, affordability, trade, and applications. The detailed measurement variables are summarized in Table 1. Some measurement variables suffered from a high proportion of missing values and were not included in the later analysis. Unfortunately, all three measurement variables for the affordability factor had high missing values, and this factor was not included in the later analysis. Since the measurement variables were in different scales, they were first standardized with a mean of zero and standard deviation of one and then averaged to extract the corresponding factor scores. 

**Table 1:** Measurement items for ICT sector performance.

ICT factors	Measurement variables
Accessibility	
	Telephone lines (per 100 people)
	Mobile cellular subscriptions (per 100 people)
	Fixed Internet subscribers (per 100 people)
	Personal computers (per 100 people)
	^a^Households with a television set (%)
Usage	
	^a^International voice traffic (minutes/person/month)
	Mobile telephone usage (minutes/user/month)
	Internet users (per 100 people)
Quality	
	^a^Population covered by mobile cellular network (%)
	Fixed broadband subscribers (% of total Internet subscribers)
	International Internet bandwidth (bits/second/person)
Affordability	
	^a^Residential fixed line tariff (US$/month)
	^a^Mobile cellular prepaid tariff (US$/month)
	^a^Fixed broadband Internet access tariff (US$/month)
Trade	
	ICT goods exports (% of total goods exports)
	ICT goods imports (% of total goods imports)
	ICT service exports (% of total service exports)
Applications	
	ICT expenditure (% of GDP)
	^a^E-government web measure index
	Secure Internet servers (per 1 million people)

^a ^Measurement items are not included in the later analysis due to high missing values.

Data for the public health indicators were extracted from the World Development Indicators database [25], which includes adolescent fertility rate, child immunization, tuberculosis case detection rate, life expectancy, and adult mortality rate. The definitions of these indicators are summarized in Table 2. Child immunization consists of two variables: immunization against DPT and against measles. Likewise, adult mortality rate contains two variables: mortality rates for females and males. All the variables were normalized, and the values of child immunization and adult mortality rate were calculated as the averages of their two measurement variables respectively.

**Table 2:** Measurement items for public health indicators.

Variables	Measurement variables
Adolescent fertility rate	The number of births per 1000 women ages 15−19.
Child immunization	The percentage of children ages 12−23 months who received vaccination against diphtheria, pertussis, and tetanus (DPT) as well as measles.
Tuberculosis case detection rate	The ratio of newly notified tuberculosis cases (including relapses) to estimated incident cases (case detection, all forms).
Life expectancy	The number of years a newborn infant would live if prevailing patterns of mortality at the time of its birth were to stay the same throughout its life.
Adult mortality rate	The probability of dying between the ages of 15 and 60 (per 1000 female adults); that is, the probability of 15-year-old dying before reaching age 60, if subject to current age-specific mortality rates between those ages.

The dataset of ICT factors and public health indicators were extracted based on five income groups defined by World Bank (ie, high-income countries, upper high-income countries, middle-income countries, lower middle-income countries, and low-income countries) and then merged based on the income groups. The final dataset contained five country categories for nine years (from 2000−2008).

### Analysis Method

The primary analysis method in this study is panel data analysis, which is an increasingly popular form of longitudinal data analysis among social and behavioral science researchers. A panel is a cross-section or group of people who are surveyed periodically over time. With repeated observations of enough cross-sections, panel analysis permits the researcher to study the dynamics of change with short time series. The combination of time series with cross-sections can enhance the quality and quantity of data in ways that would be impossible using only one of these two dimensions. Specifically, the panel data allow us to control for variables that we cannot observe or measure in each group (eg, culture) and also help to control for unobservable variables that change over time but not across entities (eg, economic development level). 

With panel data one can include variables at different levels of analysis. In this study, we could choose each country as the level of analysis, but we set the level of analysis at an aggregate level—groups of countries based on their income. The primary reason for this choice is that the missing values of all the interested variables across 200 countries result in a substantial reduction of the dataset. Following World Bank classification, all the countries are divided into five income groups: high-income group, upper middle-income group, middle-income group, lower middle-income group, and low-income group. 

The following fixed effect model is set up to explore the relationship between ICT factors and public health delivery indicators within each group of countries, as we assume that something within a country group may impact or bias the predictor or outcome variables and that this needs to be controlled in the model. The key insight is that if the unobserved variables do not change over time, then any changes in the outcome variable must be caused by influences other than these fixed characteristics. As such, once the effect of those time-invariant characteristics from the predictor variables is removed, we can assess the predictors’ net effect on outcome variables.

**Figure 2:**
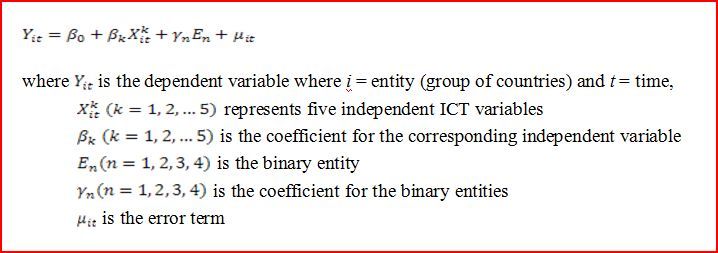
Fixed effect model.

There are two ways to build the fixed effect model, and we chose to use the one with binary variables because we could separate the association of ICT factors and income levels from public health delivery indicators. Since we have five entities (five groups of countries) to generate binary (dummy) variables, only four entities are included in the model (see Figure 2).

## Results

The data analysis was conducted in STATA. The association between the ICT factors and the five public health indicators are summarized in Tables 3 and 4. Generally, we found support for all the hypotheses. That is, the application of ICTs overall could reduce adolescent fertility rates (H1) and adult mortality rates (H5), increases the coverage of child immunization (H2) and the chances of tuberculosis detection (H3), as well as lengthens life expectancy (H4).

Second, among all the ICT factors, accessibility is the only one that is associated with improvements in all aspects of public health delivery, while the contributions from the usage, quality, and applications are negligible. This is possibly due to lack of longitudinal data or the fact that some aspects of the application of ICTs in public health are only now emerging.

**Figure 3:**
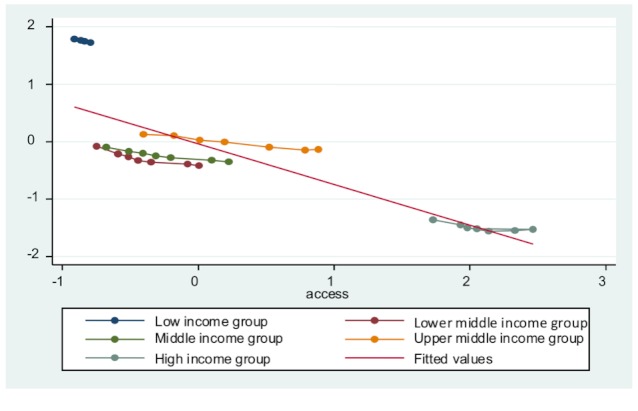
Impact of ICT accessibility on adolescent fertility rate.

**Figure 4:**
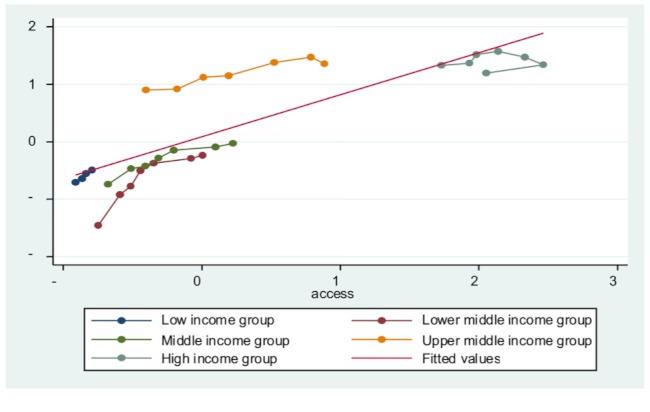
Impact of ICT accessibility on child immunization coverage.

**Figure 5:**
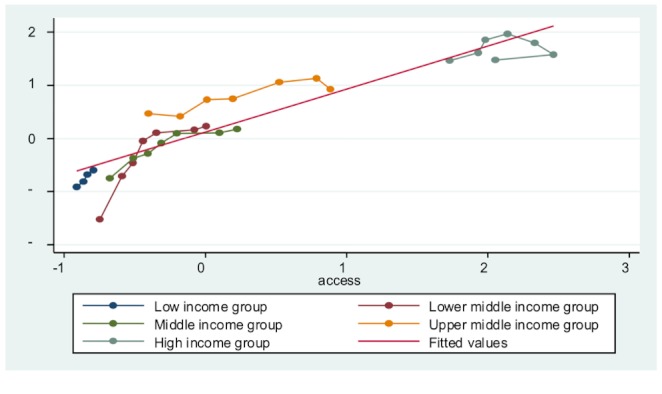
Impact of ICT accessibility on tuberculosis case detection rate.

**Figure 6:**
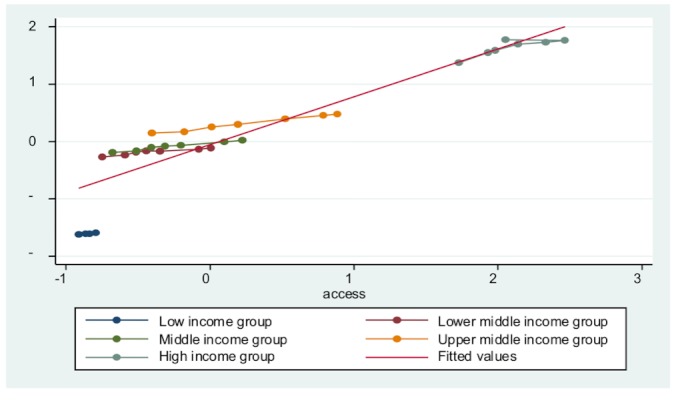
Impact of ICT accessibility on life expectancy.

**Figure 7:**
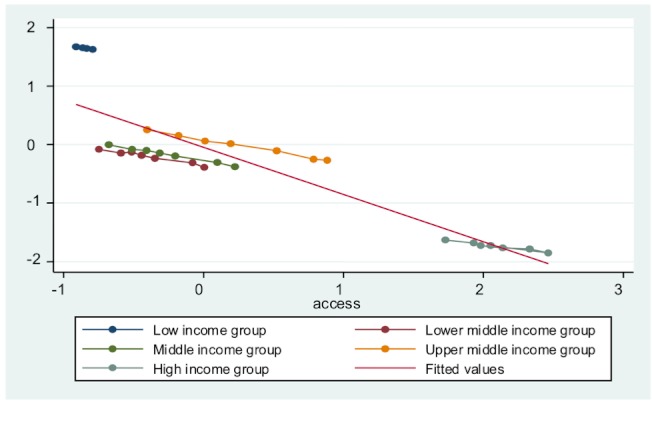
Impact of ICT accessibility on adult mortality rate.

Third, all entity-specific factors are significant in each model, indicating that countries’ economic development level does influence their public health delivery. To an extent, developed countries do have a base level of public health delivery. This is because of overall higher standard of living, better education, and less poverty. Figures 3 to 7 plot the ICT accessibility factor against each public health indicator for each group of countries. Overall, high-income countries enjoy higher ICT accessibility and better health conditions, whereas low-income countries have low accessibility and poor health conditions. Thus, wealth effect is confirmed.

**Table 3:** Associations between ICT factors and public health delivery indicators (adolescent fertility rate, child immunization, and tuberculosis detection).

Independent variables	Adolescent fertility rate		Child immunization		Tuberculosis detection	
	Coefficient	*P *value	Coefficient	*P *value	Coefficient	*P *value
Accessibility	-0.28	<.001	0.64	<.001	0.86	<.001
Usage	-0.03	.32	0.14	.16	0.20	.07
Quality	<0.001	.05	<-0.001	.21	<-0.001	.06
Trade	-0.15	.01	0.90	<.001	1.37	<.001
Application	-0.05	.15	-0.06	.61	0.04	.76
Lower middle-income countries	-1.33	<.001	-3.73	<.001	-5.13	<.001
Middle-income countries	-1.39	<.001	-2.67	<.001	-3.82	<.001
Upper middle-income countries	-1.18	<.001	-0.70	.09	-2.06	<.001
High-income countries	-1.98	<.001	-1.94	.005	-3.45	<.001
Constant	1.12	<.001	2.10	<.001	3.31	<.001
*F *test that all U_i_=0	76.86	<.001	38.51	<.001	19.05	<.001

**Table 4:** Associations between ICT factors and public health delivery indicators (life expectancy, adult mortality rate).

Independent variables	Life expectancy		Adult mortality rate	
	Coefficient	*P *value	Coefficient	*P *value
Accessibility	0.30	.03	-0.35	<.001
Usage	-0.004	.89	-0.04	.30
Quality	<-0.001	.13	<0.001	.84
Trade	-0.07	.52	0.01	.88
Application	0.14	.15	0.05	.35
Lower middle-income countries	1.54	.01	-1.72	<.001
Middle-income countries	1.57	.002	-1.63	<.001
Upper middle-income countries	1.78	<.001	-1.28	<.001
High-income countries	2.31	.01	-2.40	<.001
Constant	-1.46	.003	1.38	<.001
*F *test that all U_i_=0	113.91	<.001	38.36	<.001

## Discussion

### Key Results

First, our results indicate that overall ICT factors substantially improve a country’s public health delivery on top of the wealth effect. As mentioned previously, ICTs are not vaccines or medications or health protocols, rather ICTs are technologies that enable the processing of health information more efficiently and effectively. This has implications for health policy, particularly to developing countries.

In developing countries, lack of access to health care and inefficient health care delivery methods are the norm. Poor health care is characterized by high infant, female (pregnancy & birth mortality), and adult mortalities, low immunization rates, death from diseases, and low life expectancies. The shortage of health care is worsened by the inability of governments to devote adequate funding to their respective health care sectors, and the shortage is compounded by the multiple crises in finance, food, and energy. Unsuccessful reform efforts, combined with little funding, have left billions without the ability to tap into basic health care services. The investment in ICTs is channelized for use in health care via education, remote delivery of health care, and monitoring and tracking, for example.

Second, not all the ICT factors generate similar effects on public health indicators—some produce significant effects while others contribute little. As such, the government of a country can focus on ICT investment to achieve its specific goal in public health. For instance, ICT accessibility factors are associated with a considerable extension to life expectancy and reduced adult mortality rate. This is possibly due to the use of ICTs in the key strategies of education and preventive care enabled by ICTs. These two variables are also linked to poverty alleviation and economic development. Additionally, the access and trade factors of ICTs result in reduced adolescent fertility rates. This can be rationalized by the observation that having access to ICTs and the economic development activity of ICT trade indicate a higher level of influence of education and awareness of health consequences. In others, ICTs make the population more sophisticated in the access to health information. Likewise, access and trade improve child immunization coverage. This again indicates the fact that access to mobile and other ICTs by both the general population and health workers leads to increased awareness of immunization benefits, vaccination schedules, and follow-up processes leading to an increase in coverage. However, only access factors appear to have an impact on tuberculosis case detection rates, life expectancy, and adult mortality rates. But access is a key ICT factor, and it is not critical that every ICT factor have some bearing on public health delivery. There are possible variations in the use of ICTs across geographic locations and cultures. Additionally, life expectancy and adult mortality rates have to be examined over a very long period of time.

Finally, the analysis results also suggest that in almost all areas studied, countries in the higher-income groups have progressed further in the adoption of actions and provision of services than those in the lower-income groups. This finding is not surprising; it confirms that the “digital divide” includes eHealth. Our common goal should be, therefore, to lessen this divide with concerted action [18]. First, by investing in ICT’s targeting of public health, the delivery of public health is enhanced. Second, although the effect of wealth is acknowledged, the cumulative investment in ICTs will improve public health delivery regardless of the development stage of a country. Third, accessibility has the greatest association with positive delivery of public health vis-à-vis the other ICT factors. Therefore, enabling accessibility ought to be the top priority in ICT infrastructure investment, particularly in developing countries.

It is also important to note that the study does not show a direct causality between ICTs and public health delivery. In other words, the study does not say that by using ICTs, health improves. Rather, we conclude that there is a strong association in the relationship between certain ICTs and how public health is delivered. In this regard, the findings support the anecdotal and case studies evidence reported in the literature. Further longitudinal studies in the linkages between specific ICT applications (eg, introduction of a telemedicine project) and health indicators (eg, reduction in the number of hospital visits) may address causal relationships. But large “big data” sets are needed for this type of study.

### Conclusion

The contributions of this study are several. For one, by further studying the association between ICTs and public health indicators empirically using panel data analysis, we contribute to the sparse literature in this particular area. Note that we acknowledge the portfolio of general research that has emerged in health information technology—see special issues of JAIS [22] and JSIS [23]. The findings can help global policy makers strategize on ICT health resource allocation and invest in technologies that would maximize the population health benefits.

ICTs have the potential to radically transform the delivery of public health. There are others, but the key strategic applications are eHealth and mHealth. The findings of this study will help government officials and public health policy makers to formulate strategic decisions regarding the best ICT investments and deployment. For example, the study shows that providing accessibility ought to be a critical focus. While the use of ICTs is one among several strategies in improving global public health, the delivery of public health cannot be replaced or underplayed. The actual carrying out of immunizations and vaccinations and preventive health measures, health education, hygiene, clean water, and sanitation are vital factors in promoting public health. In the long term, better public health can improve productivity, contribute to poverty alleviation, and enhance quality of life overall.

Several research directions are possible. The emerging field of health analytics can be applied to “big health data” sets for more robust analysis resulting in richer insight and informed decision making. While most governmental and NGOs (eg, World Bank, WHO, Rockefeller Foundation) report and publish surveys, there is a paucity of quantitative analysis of the raw data, or aggregated data. The application of health analytics techniques will enrich findings. While this is a study done at the aggregate level, it is important to continue case studies of specific applications and deployments in various countries to gain insight into the sociotechnical dimensions of the role of ICTs in health care. These include social, cultural, and political aspects of what works and what does not. The lessons learned can be shared with characteristically similar countries so as to avoid costly investment mistakes. Thus, knowledge creation and sharing is important. Also, future research may continually monitor and update the variables and data sets in real time and perform cross-country and cross-regional research as well as additional longitudinal studies. Other variables and development indicators may reveal additional associations and effects. An important issue to address is the “reverse effect” or “rebound effect”. What are the negative consequences of using or overusing technology? Carpel tunnel syndrome, for instance, is symptomatic of repeated use of the keyboard. Excessive cell phone use or constant staring at electronic screens or monitors also may have adverse effects. Going beyond quantitative studies, one may research the diffusion of ICTs for public health purposes and consider additional concepts and models, such as the introduction of key “disruptive technologies” into health care, the “leap frog” effect, the alleviation of the “digital divide” with regard to health care, “local innovation”, and new health care delivery designs and models. Additionally, new resource allocation and investment models and strategies of ICTs may emerge. Their adoption and assimilation may be studied. Globally, the application of ICTs such as eHealth and mHealth is rapidly, if unevenly, proliferating. Much needs to be done to provide universal and equitable health care to all. 
